# The Hypoxia–Retinoid Axis in Idiopathic Pulmonary Fibrosis: Multifaceted Etiology and Therapeutic Potential

**DOI:** 10.3390/ijms26115302

**Published:** 2025-05-31

**Authors:** Daniel Paz-Gomez, Manuel Castillejos-López, Yair Romero, Edgar Flores-Soto, Bianca S. Romero-Martinez, Joel Armando Vázquez-Pérez, Georgina Gonzalez-Avila, Victor Ruiz, Ángeles Carlos-Reyes, Rafael Velázquez-Cruz, José Alberto Choreño-Parra, Roberto Lara-Lemus, Fausto Rojas-Duran, David Martínez Briseño, Joaquín Zuñiga, Luz María Torres-Espíndola, Arnoldo Aquino-Gálvez

**Affiliations:** 1 Laboratorio de Investigación en Enfermedades Reumáticas, Instituto Nacional de Enfermedades Respiratorias, Ismael Cosío Villegas (INER), Mexico City 14080, Mexico; danielpazgmz@gmail.com; 2 Unidad de Epidemiología Hospitalaria e Infectología, Instituto Nacional de Enfermedades Respiratorias Ismael Cosío Villegas (INER), Mexico City 14080, Mexico; mcastillejos@gmail.com (M.C.-L.); mb3david@gmail.com (D.M.B.); 3 Facultad de Ciencias, Universidad Nacional Autónoma de México (UNAM), Mexico City 04510, Mexico; yair@ciencias.unam.mx; 4 Departamento de Farmacología, Facultad de Medicina, Universidad Nacional Autónoma de México (UNAM), Mexico City 04510, Mexico; edgarfloressoto@yahoo.com.mx (E.F.-S.); biancasromero_@hotmail.com (B.S.R.-M.); 5 Laboratorio de Biología Molecular de Enfermedades Emergentes y EPOC, Instituto Nacional de Enfermedades Respiratorias Ismael Cosío Villegas (INER), Mexico City 14080, Mexico; joevazpe@gmail.com; 6 Departamento de Enfermedades Crónico-Degenerativas, Instituto Nacional de Enfermedades Respiratorias Ismael Cosío Villegas (INER), Mexico City 14080, Mexico; ggonzalezavila@yahoo.com; 7 Laboratorio de Biología Molecular, Departamento de Fibrosis Pulmonar, Instituto Nacional de Enfermedades Respiratorias Ismael Cosío Villegas (INER), Mexico City 14080, Mexico; vicoruz@yahoo.com.mx; 8 Sección de Estudios de Posgrado e Investigación, Escuela Superior de Medicina, Instituto Politécnico Nacional (INP), Mexico City 11340, Mexico; 9 Laboratorio de Onco-Inmunobiología, Departamento de Enfermedades Crónico-Degenerativas, Instituto Nacional de Enfermedades Respiratorias Ismael Cosío Villegas (INER), Mexico City 14080, Mexico; reyes_cardoso@yahoo.com; 10 Laboratorio de Genómica del Metabolismo Óseo, Instituto Nacional de Medicina Genómica (INMEGEN), Mexico City 14610, Mexico; rvelazquez@inmegen.gob.mx; 11 Formación de Posgrado, Departamento de Enseñanza, Instituto Nacional de Enfermedades Respiratorias Ismael Cosío Villegas (INER), Mexico City 14080, Mexico; choreprr@gmail.com; 12 Departamento de Biomedicina Molecular e Investigación Traslacional, Instituto Nacional de Enfermedades Respiratorias Ismael Cosío Villegas (INER), Mexico City 14080, Mexico; antonio.lara@iner.gob.mx; 13 Instituto de Investigaciones del Cerebro, Universidad Veracruzana (UV), Xalapa 91190, Veracruz, Mexico; frojas@uv.mx; 14 Laboratorio de Inmunobiología y Genética, Instituto Nacional de Enfermedades Respiratorias Ismael Cosío Villegas (INER), Mexico City 14080, Mexico; joaquin.zuniga@iner.gob.mx; 15 Laboratorio de Farmacología, Instituto Nacional de Pediatría (INP), Mexico City 04530, Mexico; 16 Departamento de Bioquímica, Facultad de Medicina, Universidad Nacional Autónoma de México (UNAM), Mexico City 04510, Mexico

**Keywords:** idiopathic pulmonary fibrosis (IPF), retinoids, all-trans retinoic acid (ATRA), hypoxia, lung regeneration, combination therapy

## Abstract

Idiopathic pulmonary fibrosis (IPF) is a progressive and lethal lung disease with limited therapeutic options. This review focuses on the role of retinoids, particularly all-trans retinoic acid (atRA), and hypoxia in the pathogenesis of IPF. Despite an established understanding of genetic and environmental factors in IPF, the interplay between retinoid signaling and the response to hypoxia remains poorly explored due to its complexity. Preclinical evidence suggests that atRA could help reduce pulmonary fibrosis by modulating TGF-β signaling pathways and epithelial-to-mesenchymal transition (EMT). Additionally, we mention other diseases where a relationship between hypoxia and retinoids has been observed. We review how hypoxia, a key factor in the progression of IPF, may influence the efficacy of retinoid therapy. Combination strategies are explored to overcome hypoxia-induced treatment resistance. Finally, we address the complex role of retinoids in lung regeneration, balancing their potential benefits against the risk of exacerbating fibrotic processes. This review suggests that retinoids have potential as a treatment or adjuvant for IPF and highlights the need for further research to elucidate the precise mechanisms of retinoid action in IPF, particularly in hypoxia.

## 1. Introduction

Disruption of cell homeostasis in the respiratory system, abnormal tissue repair caused by a genetic deficiency, and exposure to risk factors lead to a potentially lethal lung disease termed idiopathic pulmonary fibrosis (IPF) [1]. IPF is an incurable lung disease of unknown cause that is predominantly seen in people older than 65 years old, and its prevalence increases with age, suggesting a relation with aging [2,3,4,5]. It has a survival median of 2–3 years, and it is characterized for being progressive with a poor prognosis; patients show progressive dyspnea and an unproductive cough that produces restrictive disrepair with a decrease in carbon monoxide diffusion capacity, which leads to a declining quality of life [6,7].

IPF is a multifactorial polygenic disease, and several genetic polymorphisms have been identified as risk factors for the development of IPF. These include genes related to telomere integrity, surfactant protein, and Mucin 5B (MUC5B) [8,9,10], and rare genetic variants enriched in smooth muscle cells, alveolar epithelial type II (AE2) cells, and endothelial cells [11]. Approximately 20% of IPF cases are familial [11,12], but it has been suggested that specific epigenetic patterns, specially DNA methylation, histone modification, lncRNAs, and microRNA, affect endophenotypes that underlie the development of IPF [13], supporting the polygenic nature of the disease.

Environmental factors also play a role in IPF, observing an additive effect of air pollution and genetic susceptibility in its pathophysiology [14]. Some studies shown that pollution and exposure to NO_2_ could increase the risk of development and aggravate the severity of IPF, leading to an increase in mortality [14,15,16,17]. Other known risk factors are obesity, exposure to tobacco fumes in infancy, anxiety, depression, unhealthy lifestyle combined with a genetic risk, malnutrition, circadian clock dysfunction, prolonged night hypoxemia, and gastroesophageal reflux disease [18,19,20,21,22,23,24,25].

The type of immune response also has a role in IPF. It has been shown that IL-17A, the main cytokine of type 17 immunity, is able to induce EMT through the production of TGF-β, direct stimulation of fibroblasts and fibrocytes, and autophagy inhibition that otherwise would protect against lung fibrosis [26]. Furthermore, the subtype of M2 macrophages present could also be determinant in the development of IPF [27].

It is important to highlight that aging as a risk factor for IPF has recently taken interest [27,28] since it is known that it results in progressive damage in lung function, even in healthy individuals [29]. In addition, the transcriptomic data of old animals significantly correlates with IPF patients [30]. It has been proposed that dysfunction and loss of AE2 cells together with a failed regeneration contribute to IPF. Inducing lung damage leads to the expression of aging-related genes even in young mice, which suggests a synergistic effect of aging and AE2 cell lesions in the development of fibrosis [28]. Furthermore, cellular senescence has been observed in lung epithelium and mesenchymal cells of IPF patients, suggesting that senescent fibroblasts could be enough to start a progressive fibrogenic reaction in the lung [31,32].

## 2. Metabolic Changes in Idiopathic Pulmonary Fibrosis

Alterations in glycolysis, beta-oxidation, the tricarboxylic acid cycle, biliary acids, heme, and glutamate/aspartate metabolism have been found in the lungs of IPF patients [33,34]. Furthermore, mitochondrial dysfunction is observed in the alveolar epithelia of IPF patients [35] as well as metabolic heterogeneity. However, it remains unclear whether this metabolic heterogeneity drives the clinical variability seen in patients or if the reverse is true.

IPF has been broadly documented to present the altered synthesis and activity of fatty acids, cholesterol, and other lipids [36,37,38]; homeostasis of their metabolism is required to maintain the function of AE2 cells [39]. AE2 cells are key in regeneration and repair processes, but they seem to be dysfunctional in IPF, probably due to lipid metabolism alterations [40]. Accordingly, ectopic adipocyte deposits could be observed in subpleural fibrotic regions [41], and AE2 cells increase cholesterol synthesis and lipofibroblast production with aging [42]. In patients with radiation-induced fibrosis, metabolic changes have been observed that may be due to a high energetic demand in fibroblast proliferation [43]. Fatty acid oxidation is needed to obtain energy in hypoxia [44], and it could explain the increase in hypoxia-induced transcriptional factors in IPF patients [45].

## 3. Hypoxia and Progression of Idiopathic Pulmonary Fibrosis

Hypoxia is a key factor in the development and progression of IPF [45,46,47,48,49]. Hypoxia in fibroblast foci leads to a poor response to treatment in IPF patients, which is why nanoparticles have been designed to release drugs in response to hypoxia, thus improving treatment response [50]. Oxygen therapy during lung rehabilitation improves lung function and quality of life [51], and mechanical ventilation might be useful to treat acute exacerbation in patients with interstitial lung fibrosis [52], which is why hyperbaric oxygen has been proposed as treatment for lung fibrosis [53].

Progressive lung fibrosis is the result of dysfunctional tissue repair and is characterized by extracellular matrix accumulation and fibroblast proliferation, activation, and invasion. We have suggested that hypoxia-inducible factor 2α (HIF-2α), a paralog of HIF-1 and HIF-3α, is a key factor in IPF development by inhibiting lung repair or regeneration [54,55,56]; this is evidenced by its elevated expression in pulmonary fibroblasts from IPF patients, in contrast to its absence in the epithelial cell epithelium [45]. In our hypothesis, hypoxia-response pathways are needed for regeneration, but, if maintained, they could activate feedback circuits related to progression of the disease [57]. This is consistent with a recent study showing that HIF2-α activation promotes the development of aberrant epithelial cells and, thus, fibrosis progression [58]. In this work, HIF2-α inhibition attenuated pulmonary fibrosis in several models by promoting alveolar repair through alveolar epithelial cell differentiation [58]. These results suggest that HIF2-α inhibition represents a promising therapeutic strategy for IPF. It is important to note that further investigation is required, considering the heterogeneity of pulmonary fibroblasts obtained from IPF patients [54,59].

## 4. Retinoid Uptake, Metabolism, and Storage

Though the term vitamin A refers to *all-trans*-retinol (atROL), it also groups its natural derivatives and compounds with a similar biological activity. Sporn proposed the term retinoid to name all the natural and synthetic structural analogs of retinol either with its biological activity or not [60,61,62]. Since then, retinoid has been used preferentially over vitamin A, and its definition has been extended to include compounds with biological activity similar to retinol but structurally different [60,63].

**Figure 1:**
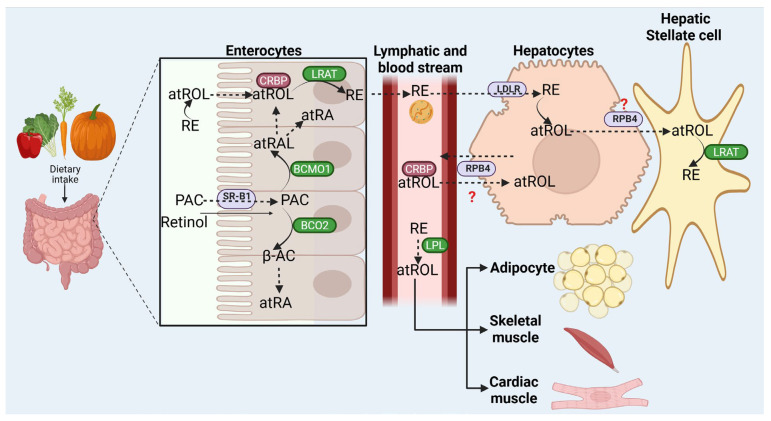
Retinoid metabolism. Retinoids are ingested in the diet and absorbed in the small intestine. Once they are transported into the enterocytes, they can travel through the lymphatic system and bloodstream in the form of all-*trans*-retinol (atROL) attached to cellular retinol binding proteins (CRBP) or albumin, while retinyl esters (REs) are incorporated into chylomicrons. Most retinoids are captured by hepatocytes, while a part of RE is hydrolyzed by lipoprotein lipase (LPL) into atROL and also taken up by extrahepatic tissues. Both RE and atROL can be stored in hepatocytes and stellate cells. PAC, provitamin A carotenoids; RE, retinyl ester; atROL, all-*trans*-retinol; SR-B1, scavenger receptor class B; CRBP, cellular retinol binding protein; BCMO1, β-carotene-15,15′-monooxygenase 1; BCO2, β,β carotene 9′,10′-dioxygenase; β-AC, β-apocarotenal; atRA, all-*trans*-retinoic acid; LRAT, lecithin:retinol acyl transferase; LPL, lipoprotein lipase; LDLR, LDL receptor; RPB4, retinoid-binding protein 4.

Retinoids cannot be synthesized de novo by animals; they have to be provided in the diet as retinol, retinyl esters (REs), or in the form of provitamin A carotenoids. Retinol and carotenoids are directly absorbed through diffusion by enterocytes in the small intestine [64], while RE must be first hydrolyzed to atROL in the intestinal lumen by non-specific pancreatic enzymes (e.g., pancreatic triglyceride lipase and cholesteryl ester lipase) or, in the mucosal cell surface, by a specific retinyl ester hydrolase, i.e., the brush border phospholipase B. On the other hand, the intestinal absorption of β-carotenes is mediated by scavenger receptor class B (SR-B1) [64]. After cellular uptake, atROL binds to cellular retinol binding proteins (CRBPs) that mediate its transport, protection, and solubilization to facilitate further enzymatic reactions (Figure 1).

In the enterocyte, carotenoids could be symmetrically or asymmetrically cleaved. The former is performed by the cytosolic enzyme β-carotene-15,15′-monooxygenase 1 (BCMO1) and produces two molecules of *all-trans*-retinal (atRAL) [65], while the asymmetrical cleavage, which yields two β-apocarotenals of different length, is carried out by enzymes such as mitochondrial β,β carotene 9′,10′-dioxygenase (BCO2) [66]. The longer β-apocarotenal could then be (i) cleaved to yield atRAL [67], (ii) oxidized to β-carotenoid acid and then processed in a β-oxidation-like reaction producing atRA [67,68], or (iii) act as signaling molecule [69,70]. The atRAL thus produced from carotenoids could be further reduced to atROL by an intestinal retinal reductase or oxidized to atRA by a retinal dehydrogenase. Both metabolites can be directly secreted to the bloodstream bound to CRBP or albumin.

atROL could then be esterified to fatty acids mainly by the enzyme lecithin:retinol acyl transferase (LRAT) [71,72]. Diacylglycerol acyltransferase 1 (DGAT1) catalyzes this reaction when atROL is not bound to a CRBP, e.g., when vitamin supplements are taken and there is an excess of atROL [73,74]. REs thus produced are packed in chylomicrons together with uncleaved carotenoid triglycerides, cholesteryl esters, and apoprotein B-48 (Figure 1) [64,75].

The nascent chylomicrons are secreted into the lymphatic ducts [76], and subsequently, they reach the blood circulation. From here, chylomicron RE could be hydrolyzed by lipoprotein lipase (LPL), and the released atROL is taken up by extrahepatic tissues such as adipocytes [77] and skeletal and cardiac muscles [78]. Nevertheless, about 75% of the REs and pro-retinoids present in chylomicrons remnants (CMRs) are captured by the liver [79]. This process is complex and involves several enzymatic reactions and retinol-binding proteins acting in an ordered way; in fact, some steps in the process of hepatic storage and the subsequent mobilization of retinoids and REs are still not totally characterized. In the liver, hepatocytes take the CMRs either by direct endocytosis or by a receptor-mediated process through the low-density lipoprotein (LDL) receptor, which recognizes with high affinity apoprotein-E (ApoE), or by the alternative receptor, i.e., LDL receptor-related protein. The REs can be hydrolyzed to produce atROL and transferred by an unclear mechanism to the hepatic stellate cells (HSCs), where atROL is esterified again by LRAT and stored inside cytoplasmic lipid droplets [80]. The identity of the RE hydrolases responsible for releasing atROL from REs stored in lipid droplets remains uncertain; however, it is suggested that four enzymes are involved at least in vitro: esterase-10 (ES-10), LPL, PLRP2, and hormone sensitive lipase (HSL).

On the other hand, the mechanisms involved in the transport of retinoids between hepatocytes and HSCs are unclear, but it is known that retinoid-binding protein 4 (RPB4) is the natural ligand for retinoids in hepatocytes [81]. RPB4 is synthetized in the endoplasmic reticulum as apoRPB4 and accumulates until atROL is available to bind it, and then it is secreted from hepatocytes into the bloodstream. RPB4 is also expressed in different extrahepatic tissues [82,83]; in fact, several reports suggested that extrahepatic RPB4 is responsible for delivering atROL from tissues (adipose tissue, kidneys, retinal pigment epithelium, testes, brain, and lungs) into the liver [83]. *Rpb4* −/− mice accumulate retinoids in HSCs, but the retinoids cannot be mobilized [84], while the knockout of Stra6, the tissue receptor for RPB4, is lethal [85]. These findings show that when there is a deficiency in RPB4, the liver is able to package and deliver retinoids in VLDL, highlighting the importance of the regulatory lipoproteins such Apo C-II and Apo E in the transport of retinoids to the tissues. In this sense, under these conditions, the roles of LPL and Stra6 gain more relevance (Figure 1).

Retinoids reach the lungs through the bloodstream in several ways: (i) REs and carotenoids in chylomicrons, chylomicron remnants, VLDL, LDL, and HDL; (ii) atROL bound to extracellular retinol binding protein 4 (RBP4); (iii) atRA bound to albumin; and (iv) β-glucuronides of atROL and atRA. Retinoids then could suffer reactions similar to those that occur in the enterocyte, leading to the formation of REs, atRAL, and atRA. REs are stored mainly in lipid droplets [86], and their accumulation in the lungs is enhanced when RA or RA analogs are provided in the diet with atROL [87]. atRAL is produced by retinol dehydrogenase (RDH) or alcohol dehydrogenase (ADH) and can be further oxidized to atRA by retinaldehyde dehydrogenases (RADHs).

## 5. Retinoic Acid Receptors

atRA is the main retinoid signaling molecule, and it exerts its function by migrating to the nucleus bound to either cellular retinoic acid-binding protein 2 (CRABP2) or fatty acid-binding protein 5 (FABP5). CRABP2-bound atRA binds to one of the three retinoic acid receptor (RAR) isotypes (i.e., α, β, and γ), which can further bind to Retinoid X receptors (RXR) to promote the transcription of target genes with a retinoic acid response element (RARE) in its sequence. FABP5-bound atRA binds to the transcription factor peroxisome proliferator-activated receptors beta/delta (PPARβ/δ) [88]. RAR and PPARβ/δ have opposing roles: RAR has anticarcinogenic activity by promoting cell differentiation, cell cycle arrest, and apoptosis, while PPARβ/δ protects from apoptosis and induces cell proliferation [88,89]. atRA binds mainly to RARs, but binding to a specific transcriptional factor might be tissue-specific and depend on the concentration of atRA and expression levels of CRABP2, FABP5, RARs, and PPARβ/δ [88]. Excess atRA is degraded by enzymes of cytochrome P 450 subfamily 26 (CYP26).

Other atRA isomers are also present in the body, i.e., 9-*cis*RA, 13-*cis*RA, 11-*cis*RA, and 9,13-*dicis*RA, but atRA is the main biologically active isomer. 13-*cis*RA and 9,13-*dicis*RA levels are equal or greater than atRA, but they cannot bind to nuclear retinoid receptors, while 9-*cis*RA can bind to either RARs or RXRs but has been found at very low levels in the human body except in the liver or in plasma following liver consumption [90]. The fact that unlike atRA 9cRA can directly bind to RXR suggests an additional role for 9cRA in promoting the transcription of genes with RXR responsive elements (RXRE). 13-*cis*RA could exert its function by isomerizing to atRA and acting as a reservoir.

atRA and other retinoids could also bind to retinoic acid-related orphan receptors (ROR) β and γ to inhibit their transcriptional activity in some neuronal cell lines when tested in a GAL4/UAS assay [91]. RORγ and its related receptor RORα bind and are negatively regulated by cholesterol and other oxysterols. Furthermore, the synthesis of atRA is also regulated by cholesterol and oxysterols through the upregulation of RADH [92]. Unlike RARs, this family of receptors binds DNA as monomers, and they are unable to bind to RXRs [93]. Their activity could be antagonized by REV-ERB nuclear receptors, which bind to the same ROR response elements (ROREs) in the DNA [93,94]. By regulating the transcription of their target genes, RORs are involved in immunity, circadian cycle regulation, embryonic development, cell differentiation, and metabolism [93,94], and it has been shown that RORα, which is also expressed in lungs, has a role in the development of hepatic and gut fibrosis [95,96].

## 6. Retinoid Receptors and Hypoxia

RORα receptor plays a role in hypoxia response and the regulation of physiological and pathological processes, including neuroprotection and cardiovascular function [91,92,93,94,95,96,97,98,99]. In the brain, RORα protects neurons and astrocytes from hypoxia- and stress-induced apoptosis, possibly by downregulating HIF-1α [99]. In cancer, RORα interaction with POU6F1 inhibits HIF1A transcription, thus suppressing the proliferation of adenocarcinoma cells in the lung [100]. Furthermore, hypoxia regulation of HIF-1α affects the function of regulatory T cells that express RORγt by modulating IL-10 production and immune response [101]. Then, RORα modulates the cell response to hypoxia and regulates key processes in diverse pathologies, highlighting its potential as a therapeutic target.

**Figure 2:**
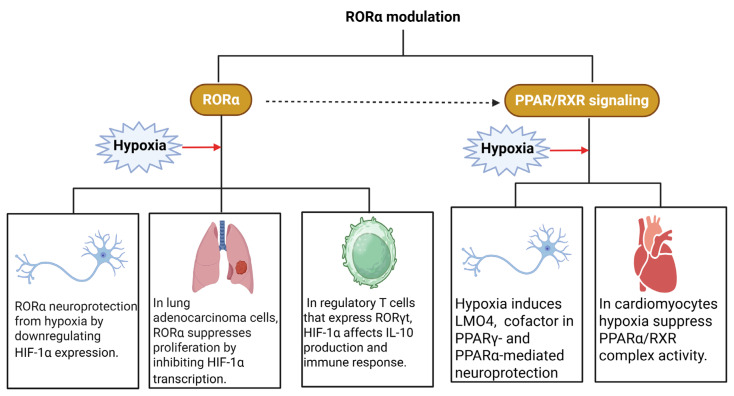
Retinoid receptors and hypoxia. The activation of retinoic acid-related orphan receptor α (ROR α) ameliorates the deleterious mechanisms induced by hypoxia, while the peroxisome proliferator-activated receptor (PPAR) and Retinoid X receptor (RXR) signaling pathways are affected by hypoxia.

Hypoxia also affects the expression and function of PPAR and retinoid signaling. In cerebrovascular events, hypoxia induces LMO4 expression, a cofactor needed for PPARγ- and PPARα-mediated neuroprotection [102]. In cardiac myocytes, hypoxia suppresses the activity of the PPARα/RXR complex, thus regulating fatty acids metabolism [103,104]. Both retinoids and hypoxia induce expression of lipid transporters ABCA1 and ABCG1 needed for lipid homeostasis and for atherosclerosis prevention [105] (Figure 2).

## 7. Retinoids in Lung Regeneration and Fibrosis: A Delicate Balance

Lung development during embryogenesis is highly susceptible to changes in atRA levels, and its deficiency leads to lung hypoplasia, agenesis, or aplasia. The lung bud primordium is unable to form from lung progenitor cells in atRA-deficient foreguts due to low Fibroblast Growth Factor 10 (FGF10) levels. Downregulation of FGF10 synthesis is achieved by the (i) hyperactivation of TGF-β signaling and (ii) overexpression of the Wnt pathway inhibitor DKK1 [106].

Lung regeneration involves activation of progenitor cells through several molecular pathways that work together to replace damaged cells, thereby restoring the integrity of the respiratory system. In adult tissues, these progenitor cells have the ability to self-renew and generate different types of lung cells, including alveolar epithelial cells responsible for gas exchange and bronchial cells that maintain airway integrity. Under normal conditions, the lung appears to be largely quiescent, with the ability to respond to injury primarily through the proliferation and differentiation of progenitor cells resident in the pulmonary epithelium [107]. Regeneration could recapitulate development depending on the type of injury and also involves the modulation of growth signals and transcription factors that coordinate cell cycle entry and differentiation. As this process is intricate and highly regulated, we review the role of retinoic acid and receptors in its modulation.

Since the 1990s, studies by Massaro and Massaro have established that atRA is involved in postnatal lung regeneration [108]. It has even been shown that it may be capable of restoring regeneration in pathologies such as emphysema in animal models [109,110]. atRA promotes AE2 cell proliferation, inhibits apoptosis, and induces differentiation into AE1 cells [111]. Furthermore, reservoirs of retinoic acid have been described in interstitial cells, which are stored in the alveolar wall, increasing the expression of CRPB 1 and associated with the formation of new alveoli [112].

Alveolar epithelium regeneration is beginning to be understood thanks to the discovery of the stem cells involved [113,114]. It has been proposed that a subpopulation of Wnt-responsive AE2 cells and fibroblasts are responsible for recovering the alveolar epithelium. This interconnection between epithelial cells and fibroblasts drives the capacity to support the alveolar niche and differentiation. It is important to note that, despite the clear association between atRA and regeneration, the precise mechanisms have yet to be fully determined. For instance, in both mouse and human organoids, direct treatment with atRA resulted in smaller organoids with reduced differentiation, while the inhibition of atRA led to organoid growth and differentiation through the activation of the YAP and FGF pathways [115].

Single-cell RNA sequencing (RNA-Seq) has revolutionized our understanding of cellular diversity by enabling the identification of previously unrecognized cell populations and providing new insights into the complexities of normal epithelial and mesenchymal cells. This technique has specifically established that lipofibroblasts, endothelial cells, and alveolar epithelial cells are capable of capturing retinoids [116]. In the context of tissue regeneration, the interstitial space plays a critical role, with retinoic acid signaling regulating the activation of fibroblasts and/or myofibroblasts through FGF pathways, particularly the PDGF-α receptor pathway, which is crucial for niche formation [117,118,119,120,121]. Furthermore, key signaling pathways involved in development, such as TGFβ and Wnt, also play an active role in this process [122,123].

In the case of IPF, the pathogenesis of the disease has been associated with the aberrant response of epithelial cells and excessive extracellular matrix (ECM) secretion by fibroblasts. Additionally, atRA influences the regulation of pathways involved in the synthesis and degradation of ECM proteins, such as collagen, laminin, and fibronectin [124]. The epithelial–mesenchymal interactions, similar to those observed during tissue regeneration, are fundamental to this pathology. There is an overlap of signaling pathways regulated by atRA, including those involving FGF in fibroblasts, as well as TGF-β and Wnt signaling in both epithelial and mesenchymal cells. It is important to highlight that, while normal lung regeneration is efficient in repairing mild damage, aging, and epigenetic changes in IPF, it can be disrupted, leading to impaired regeneration or pathological changes. In fact, RNA-Seq studies have demonstrated the emergence of subpopulations of epithelial and mesenchymal cells that contribute to the progression of the disease [125]. Using the lung organoid model, it has been discovered that AE2 cells may have intermediate transition states associated with aging, cellular senescence, TGF-β, and HIF1 [126]. Particularly, it has been demonstrated in aging models that atRA indirectly induces reciprocal signaling of PDGFA, which is essential for establishing the fibroblast niche that supports the differentiation and repair of alveolar epithelial cells [119,127].

These findings suggest a potential strategy to influence this pathogenesis; however, this perspective must incorporate the bivalent potential of regeneration. Therefore, it can be inferred that atRA signaling, through its regulation, is indirectly involved in both regeneration and IPF and potentially in the associated metabolic alterations [57]. Understanding epithelial–mesenchymal interactions is crucial for unraveling the pathogenesis of IPF, particularly in the formation of the histological pattern of usual interstitial pneumonia (UIP), a hallmark of the disease. Recent studies suggest that UIP could be considered a distinct diagnostic entity, highlighting the importance of these interactions in both the diagnosis and potential treatment strategies for IPF [128].

## 8. All-Trans Retinoic Acid in Lung Fibrosis

As mentioned earlier, ADH1B has an important role in retinoid metabolism by catalyzing the conversion of atROL in atRA. Downregulation of ADH1B has been observed in some types of cancer, e.g., gastric, colorectal, and lung, where the reduction of atRA contributes to alterations in cell proliferation and death [129,130,131]. Furthermore, in colon cancer, loss of ADH1B in cancer-associated fibroblasts is linked to an increase in the tumor-promoting cytokine IL-6 [132]. Moreover, ADH1B expression is regulated by bile acids through the FXR receptor, which links retinoids and bile acid metabolisms [133]. Finally, ADH1B expression levels decrease with age [134].

atRA inhibits the radiation-induced proliferation mediated by IL-6 of a human embryonic lung fibroblastic cell line transformed by SV40 (W138VA-13) and IMR-90 cells, also derived from fetal lung fibroblasts. Likewise, IL-6 levels were reduced in the supernatants of irradiated cells treated with atRA [135]. The same group published in 2006 two models of lung fibrosis induced by exposing mice to Bleomycin (BLM) and radiation. In both models, intraperitoneal administration of atRA increased the overall survival rate and attenuated the increase in IL-6, TGFβ1, and collagen AI mRNA levels [136].

Xiaodong et al. (2013) reported that atRA attenuated lung fibrosis in a BLM model in rats by regulating TGBβ1/Smad3 in a concentration-dependent manner [137]. In the same way, atRA reduced the expression of EMT molecules present in lung fibrosis, such as α-SMA and E-cadherin [137].

Since the last century, retinoids have been used as immunomodulators and regulators of fibroblast collagen production. Fibroblasts derived from normal lung cultured in the presence of TGFβ1 showed an increase in the production of type I and III collagen. However, when atRA was added to these cultures, collagen production was inhibited. That regulation is mediated by nuclear retinoic acid receptors [138]. More recent studies in A549 cells pre-stimulated with TGFβ1 showed that atRA completely inhibited the phosphorylation of Smad2/3 (pSmad2/3) [139].

**Figure 3:**
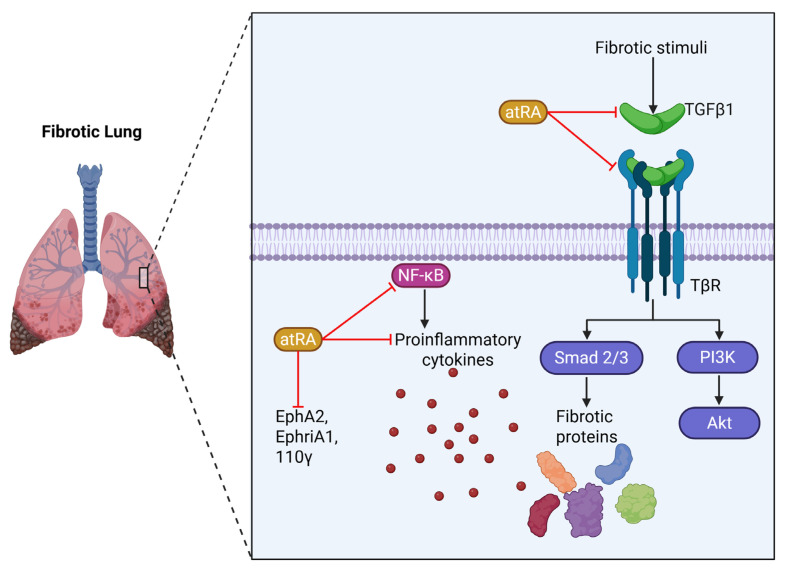
Retinoids and fibrosis. A fibrotic stimulus can upregulate transforming growth factor beta 1 (TGFβ1), which binds to its receptor and activates the phosphatidylinositol 3-kinase (PI3K) and serine/threonine-protein kinase (Akt) signaling pathways, as well as the Smad 2/3 signaling pathway, which can induce the production of fibrotic proteins. The all-*trans*-retinoic acid (atRA) can inhibit the TGFβ1 signaling pathway and downregulate the expression of EphA2, EphriA1, PI3K 110γ, proinflammatory cytokines, and nuclear factor kappa β (NF-κβ).

The development of lung fibrosis in BLM-treated rats was associated with low levels of RE, α-tocopherol, and vitamin D3 [140]. In mice treated with BLM, atRA attenuated the upregulation of IL-17A, IL-10, IL-6, EphA2, EphriA1, PI3K 110γ, Akt, IL-6, TNF-α, and TGFβ1, which reduced pulmonary fibrosis and significantly alleviated lung fibrosis [141,142] (Figure 3).

More recently, a research group demonstrated the interplay between atRA and hedgehog signaling (Hh). Rats instilled with intratracheal BLM were treated with atRA and Forskolin (FSK), an inhibitor of Hh signaling, which synergistically reversed the effect of BLM-induced lung fibrosis. FSK and atRA ameliorated oxidative stress and inflammation, reduced TGF-1 levels, and reversed the effect on the expression of Ptch-1, Smo, and Gli-2. Finally, FSK inhibited the Hh pathway and activated protein kinase A (PKA), which is involved in the phosphorylation of RAR/RXR, a key factor in retinoid receptor activation [143].

BLM-treated rats have an increase in TGF-β1/Smad, PI3K/Akt/mTOR, and NF-κB pathways, resulting in the development of lung fibrosis. Retinoids attenuated lung fibrosis mainly by inhibiting the inflammatory response through downregulating the expression of NF-κB and by inhibiting the release of the downstream cytokines TNF-α, INF-γ, and IL-13. Inhibition of fibrosis occurs via downregulation of the TGFβ/Smad signaling pathway in lung tissue (Figure 3) [144]. The observed effects of retinoids in IPF are summarized in Table 1.

## 9. Hypoxia–Retinoid Interaction in Disease

In the tumor microenvironment, hypoxia promotes therapy resistance and cancer progression, as can be seen in ductal and hepatocellular carcinomas [145,146,147]. Retinoids such as atRA and fenretinide have shown potential as novel cancer therapies by promoting a favorable epithelial phenotype and reducing cancer stem cells [145], but their efficiency is also limited by hypoxia [148]. However, the combination of retinoids with PPARγ and RXR agonists in thyroid cancer [149] and of atRA with antiangiogenic therapy in breast cancer [150], as well as with the restoration of proteins needed for retinoid signaling such as RBP1 [151], are promising strategies to enhance the efficiency of cancer therapy in hypoxia-induced resistance.

Hypoxia plays a crucial role promoting tumor growth in the glioblastoma through upregulating HIF-1α, as shown by the downregulation of GRIM-19 [152]. Hypoxia-reducing strategies, such as the use of the carotenoid crocetin, have shown therapeutic potential [153,154]. Retinoids have a dual effect on glioma angiogenesis by stimulating the formation of blood vessels at low doses and blocking their formation at higher doses, which could induce differentiation and apoptosis [155,156]. In addition, atRA can revert the stem cell-like properties induced by hypoxia in multiple myeloma, suggesting its usefulness for tumor progression in low-oxygen conditions [157].

In leukemia, hypoxia has a complex role by influencing differentiation and treatment resistance. Retinoids modulate leukemic cell differentiation, often through the HIF pathway [158,159,160]. atRA, mixed with other agents, has shown efficiency in the treatment of high-risk acute promyelocytic leukemia (APL) [161]. However, hypoxia might induce resistance to retinoids such as fenretinide in acute lymphocytic leukemia (ALL) [162]. Inhibition of HIF-1α, e.g., by using EZN-2208, could be combined with atRA to eradicate leukemia-initiating cells [163]. In addition, hypoxia might potentiate As_2_O_3_-induced differentiation in APL through HIF-1α [164]. The thyroid hormone, through activation of heterodimer RXR/TR, can also upregulate HIF-1α [164].

In neuroblastoma, intermittent hypoxia promotes an aggressive and undifferentiated phenotype through HIF-1α and HIF-2α, thus promoting resistance to retinoid therapy [165,166]. However, the inhibition of HIF-1α and HIF-2α combined with atRA induces differentiation and senescence, suggesting a promising therapy [166]. Furthermore, treatment with atRA and demethylating drugs restores sensitivity to retinoid therapy and activates HIF-2α as a tumor suppressor [167].

In the myocardium, myocardial ischemia–hypoxia has a dual role: it causes oxidative stress and apoptosis, thus damaging cardiac cells [168,169,170], and modulates retinoid activity, which influences the heart damage response. Activation of RORα and RXR by retinoids protects from hypoxia/reoxygenation injury [168,170]. In contrast, a local increase in retinoids in the infarcted area worsens the prognosis [171,172]. The modulation of retinoid uptake and RAR signaling together with HIF-1α regulation are potential therapies to mitigate myocardial damage induced by hypoxia [171,173,174].

In the kidneys, hypoxia induced by ischemia/reperfusion or vitamin A deficiency plays a crucial role in kidney damage and renal anemia by affecting the expression of protecting factors such as RORα and erythropoietin synthesis [175,176,177]. Retinoids, particularly atRA, have a protective effect by counteracting the harmful effects of hypoxia, increasing cell survival, downregulating proinflammatory and pro-fibrotic factors, and regulating the genetic expression of LMX1B, prohibitins, and components of the renin-angiotensin–aldosterone system [178,179,180,181]. In addition, both hypoxia and atRA induce HIF-1α y RARβ expression, suggesting a link between retinoid signaling and hypoxia response in kidney protection [182,183,184]. However, in clear cell renal carcinoma, both hypoxia and vitamin A deficiency activate ATF4 signaling, thus contributing to tumor progression [185]. On the other hand, in kidney cell carcinoma, the retinoid response depends on VHL function, suggesting a potential therapeutic marker [186].

## 10. Conclusions

Hypoxia has a key role in progression and therapy resistance in several types of cancer and in myocardial and kidney damage. Hypoxia, a common component in many pathologies, is intimately associated with retinoid signaling. Retinoids like atRA and fenretinide have the potential to counter the negative effects of hypoxia by promoting cell differentiation, lowering cancer stem cells, and protecting against tissue damage. However, their efficiency is limited by hypoxia and, in some cases, might even have harmful effects. The combination of retinoids with other therapies, e.g., PPARγ/RXR agonists, antiangiogenic drugs, HIF pathway inhibitors, demethylating agents, and the recovering of key proteins in the retinoid pathway, are promising strategies to overcome hypoxia-induced resistance, hence improving therapy efficiency. Furthermore, the regulation of retinoid signaling and hypoxia response through PPAR/RXR and RORα receptors is emerging as a relevant therapy in several pathologies.

Likewise, atRA has shown promise as a therapy for IPF by inhibiting fibroblast proliferation, decreasing inflammation, and reducing collagen deposition in preclinical trials. Its efficiency is based on the regulation of key pathways like TGFβ1/Smad3 and on the reversal of EMT. However, like other pathologies, hypoxic conditions in the microenvironment could also limit atRA efficiency. By promoting therapy resistance and altering retinoid signaling, hypoxia might reduce the ability of atRA to revert fibrosis. Nevertheless, the combination of atRA with other therapies like Hh signaling inhibitors or antioxidants could potentially lead to better clinical outcomes for IPF patients.

The presence of different retinoid nuclear receptors in the cells might explain the opposite role of retinoids depending on their expression and the cell type where they are present, e.g., the anticarcinogenic activity of RAR and the cell proliferation activity of PPARβ/δ [88]. Furthermore, the presence and relative abundance of different retinoids, such as other atRA isomers, could also account for the different effects [90]. This must be studied to better understand their role in fibrosis.

Some things to take into consideration before administering retinoids include the reported gender-related differences in topically administered retinoids [187], though there are still no studies on oral administration. However, given that there are differences in retinoid serum concentration between genders [188], a differential response should be considered. Furthermore, retinoids are contraindicated during pregnancy and while breast-feeding due to their teratogenic properties, and the initiation of contraception in conjunction should be considered when used as a therapy in women of childbearing potential. Other retinoid contraindications, such as an allergy to retinoids or hypervitaminosis A, should be contemplated before starting a treatment [189].

In summary, atRA has noteworthy potential for IPF treatment, but further research is required to better understand and overcome hypoxia-induced resistance to develop more efficient therapies.

## Figures and Tables

**Table 1:** Observed effects of retinoids in lung fibrosis.

Model	Retinoid	Observed Effects	References
Human embryonic lung fibroblastic cell	atRA	Reduced the increment of IL-6 levels	[135]
LF-BLM in mice	atRA	Increased the overall survival rate and attenuated the increase in IL-6, TGFβ1, and collagen AI mRNA levels	[136]
LF-BLM in rats	atRA	Reduced the expression of EMT molecules (α-SMA and E-cadherin)	[137]
Fibroblasts derived from normal lung	atRA	Collagen production was inhibited by nuclear retinoic acid receptor activation	[138]
A549 cells	RA	Completely inhibited the phosphorylation of Smad2/3	[139]
LF-BLM in rats	atRA	Attenuated in the expression of IL-17A, IL-10, IL-6, and TGFβ1	[140,142]
LF-BLM in mice	atRA	Attenuated the upregulation of EphA2, EphriA1, PI3K 110γ, Akt, IL-6, and TNF-α	[141]
LF-BLM in rats	atRA	Ameliorated oxidative stress and inflammation, reduced TGF-1 levels, and reversed the effect on the expression of Ptch-1, Smo, and Gli-2 expression	[143]
LF-BLM in rats	carotene	Downregulation of the TGFβ/Smad signaling pathway via downregulation of TGFβ1, Smad2/3, and collagen I in lung tissue and by inhibiting the release of the downstream cytokines TNF-458 α, INF-γ, and IL-13	[144]

BLM, Bleomycin; atRA, all-trans retinoic acid, LF-BLM; lung-fibrosis-induced BLM model in mice.

## Data Availability

Not applicable.

## Abbreviations

The following abbreviations are used in this manuscript:

ADH	alcohol dehydrogenase
AE2	epithelial type II cells
Akt	serine/threonine-protein kinase
ApoE	apoprotein-E
atRAL	all-trans-retinal
atRA	all-trans-retinoic acid
atROL	all-trans-retinol
BCO2	β,β carotene 9′,10′-dioxygenase
BCMO1	β-carotene-15,15′-monooxygenase 1
BLM	Bleomycin
CMR	chylomicrons remnants
CRABP2	retinoic acid-binding protein 2
CRBP	cellular retinol binding proteins
CYP26	cytochrome P 450 subfamily 26
DGAT1	diacylglycerol acyltransferase 1
ECM	extracellular matrix
EMT	epithelial–mesenchymal transition
FABP5	fatty acid-binding protein 5
FGF	fibroblast growth factor
FGF10	Fibroblast Growth Factor 10
FSK	Forskolin
Hh	hedgehog signaling
HIF	hypoxia-inducible factor
HSL	hormone sensitive lipase
HSC	hepatic stellate cells
IPF	idiopathic pulmonary fibrosis
LDLR	LDL receptor
lncRNAs	long non-coding RNAs
LPL	lipoprotein lipase
LRAT	lecithin:retinol acyl transferase
MUC5B	Mucin 5B
PI3K	phosphatidylinositol 3-kinase
PPARβ/δ	peroxisome proliferator-activated receptors be-ta/delta
RADH	retinaldehyde dehydrogenases
RARE	retinoic acid response element
RBP4	retinoid-binding protein 4
RDH	retinol dehydrogenase
RE	retinyl esters
ROR	retinoic acid-related orphan receptors
RORE	ROR response elements
RNA-Seq	single-cell RNA sequencing
RXR	Retinoid X receptors
RXRE	RXR responsive elements
SR-B1	scavenger receptor class B
TGF-β	transforming growth factor-beta
TRF	tocotrienol-rich fraction
UIP	usual interstitial pneumonia
